# Editorial

**DOI:** 10.1120/jacmp.v8i4.2796

**Published:** 2007-11-21

Ten years ago, a few ACMP members began discussing how the new communication medium, the internet, could be used to publish a peer‐reviewed clinical medical physics journal. Acting with a lot of faith, the ACMP Board of Chancellors pledged the resources of the ACMP on the promise the journal would be successful; publication began just two years later. Although a few internet journals were already flourishing by 2000, the JACMP was one of the very first open‐access journals in Medicine and Radiology. Despite some stumbling, we have completed 8 years of publication and the journal now has an international presence. This is my last Editorial as Editor‐in‐Chief, and with it, I complete four rewarding years and sixteen issues of service.

The internet was an exciting new frontier 10 years ago, and it represented a new way of thinking about the communication of scholarly ideas. I see another such frontier just emerging, and I want to be a part of it. Today, encyclopedias, operating systems, news stories and many other items are produced through mass collaboration. I believe that the medical physics community needs a Wikipedia‐type solution to the problem of expanding knowledge and information. It seems to me that the JACMP could be a large part of that effort since the ownership of the articles in the JACMP remains with the author, and the information in those articles could be contributed to such a collaborative effort. Maybe ten years from now, we will have such a resource thanks to the generosity and integrity of the medical physics community.

I would like to welcome several new Associate Editors to the JACMP: Patrick Cadman, Marco Carlone, Nathan Childress, Lawrence Court, and John Gibbons. I also want to welcome the new Editor‐in‐Chief of the JACMP, my very good friend and colleague, George Starkschall. Let me offer my very best wishes and congratulations to George as he takes the JACMP to the next level. Finally, I want to thank you, the readers of the JACMP for making us part of your professional life and community.

**The Journal of Applied Clinical Medical Physics**

Michael D. Mills, PhD

**Table:** 

**Editorial Board 2007**	
Editor‐in‐Chief	Michael Mills
Editor‐in‐Chief (beginning 2008)	George Starkschall
Deputy Editor‐in‐Chief	Timothy Solberg
**Associate Editors**	
Radiation Oncology Physics	Nzhde Agazaryan, Salahuddin Ahmad
	John Antolak, Patrick Cadman
	Marco Carlone, Nathan Childress
	Lawrence Court, Jianrong Dai
	B. Gino Fallone, John Gibbons
	Benjamin Nelms, Matthew Podgorsak
	Nikos Papanikolaou, Chester Ramsey
	William Salter, Mehrdad Sarfaraz
	Frank Van den Heuvel, Lu Wang
	Albert Zacarias, Ronald Zhu
Medical Imaging	Geoffrey Clarke, William Pavlicek
Radiation Measurements	Larry DeWerd
Radiation Protection & Regulations	James Rodgers
Nonionizing Topics	Edwardo Moros
Other Topics	Timothy Solberg
Book/Media Reviews	Michael Schell
Editorials	~
**Journal Production Team**	
Proofreader	Heather Shand
Copy Editor	Ann Fothergill‐Brown
Layout Editor	Laura Shand
Manuscript Manager	Bonnie Ratcliff

